# Prospective evaluation of the cardiac safety of HER2-targeted therapies in patients with HER2-positive breast cancer and compromised heart function: the SAFE-HEaRt study

**DOI:** 10.1007/s10549-019-05191-2

**Published:** 2019-03-09

**Authors:** F. Lynce, A. Barac, X. Geng, C. Dang, A. F. Yu, K. L. Smith, C. Gallagher, P. R. Pohlmann, R. Nunes, P. Herbolsheimer, R. Warren, M. B. Srichai, M. Hofmeyer, A. Cunningham, P. Timothee, F. M. Asch, A. Shajahan-Haq, M. T. Tan, C. Isaacs, S. M. Swain

**Affiliations:** 1 Georgetown Lombardi Comprehensive Cancer Center, Georgetown University Medical Center, 4000 Reservoir Road NW, 120 Building D, Washington, DC 20057-1400 USA; 2 MedStar Heart & Vascular Institute, Washington, DC USA; 3 Department of Biostatistics, Bioinformatics & Biomathematics, Georgetown University Medical Center, Washington, DC USA; 4 Department of Medicine, Weill Cornell Medical College, New York, NY USA; 5 Department of Medicine, Memorial Sloan Kettering Cancer Center, New York, NY USA; 6 The Johns Hopkins University School of Medicine, Baltimore, MD USA; 7 The Johns Hopkins University Sidney Kimmel Comprehensive Cancer Center, Baltimore, MD USA; 8 Washington Cancer Institute, MedStar Washington Hospital Center, Washington, DC USA; 9 AstraZeneca, Gaithersburg, MD USA; 10 Department of Cardiology, MedStar Georgetown University Hospital, Washington, DC USA; 11 MedStar Health Research Institute, Hyattsville, MD USA

**Keywords:** HER2-targeted therapy, Cardiac dysfunction, Cardiac safety, Carvedilol, Breast cancer

## Abstract

**Purpose:**

HER2-targeted therapies have substantially improved the outcome of patients with breast cancer, however, they can be associated with cardiac toxicity. Guidelines recommend holding HER2-targeted therapies until resolution of cardiac dysfunction. SAFE-HEaRt is the first trial that prospectively tests whether these therapies can be safely administered without interruptions in patients with cardiac dysfunction.

**Methods:**

Patients with stage I–IV HER2-positive breast cancer candidates for trastuzumab, pertuzumab or ado-trastuzumab emtansine (TDM-1), with left ventricular ejection fraction (LVEF) 40–49% and no symptoms of heart failure (HF) were enrolled. All patients underwent cardiology visits, serial echocardiograms and received beta blockers and ACE inhibitors unless contraindicated. The primary endpoint was completion of the planned HER2-targeted therapies without developing either a cardiac event (CE) defined as HF, myocardial infarction, arrhythmia or cardiac death or significant asymptomatic worsening of LVEF. The study was considered successful if planned oncology therapy completion rate was at least 30%.

**Results:**

Of 31 enrolled patients, 30 were evaluable. Fifteen patients were treated with trastuzumab, 14 with trastuzumab and pertuzumab, and 2 with TDM-1. Mean LVEF was 45% at baseline and 46% at the end of treatment. Twenty-seven patients (90%) completed the planned HER2-targeted therapies. Two patients experienced a CE and 1 had an asymptomatic worsening of LVEF to ≤ 35%.

**Conclusion:**

This study provides safety data of HER2-targeted therapies in patients with breast cancer and reduced LVEF while receiving cardioprotective medications and close cardiac monitoring. Our results demonstrate the importance of collaboration between cardiology and oncology providers to allow for delivery of optimal oncologic care to this unique population.

**Electronic supplementary material:**

The online version of this article (10.1007/s10549-019-05191-2) contains supplementary material, which is available to authorized users.

## Introduction

The initial trials of trastuzumab for metastatic breast cancer revealed increased risk of cardiac toxicity with rates of left ventricular ejection fraction (LVEF) decline ranging from 3 to 27% [1]. Since then, subsequent trials leading to the approval of human epidermal growth factor receptor 2 (HER2)-targeted therapies have employed stringent cardiovascular eligibility criteria, cardiac monitoring schema, and early stopping rules largely based on LV function assessed by LVEF [2–8]. In the adjuvant trastuzumab clinical trials, up to 18% of patients had asymptomatic declines in LVEF and in one study 19% discontinued trastuzumab, although the observed rates of clinical heart failure (HF) were low (0–4.1%) [9–12]. Current Food and Drug Administration (FDA) recommendations for trastuzumab, pertuzumab and ado-trastuzumab emtansine (T-DM1) limit their use to patients whose LVEF prior to treatment exceeds 50% or 55% and advise dose delay or discontinuation in the setting of LVEF decline during treatment [13–16]. The potential impact of withholding or delaying HER2-targeted therapies on oncologic outcomes is of concern, given that all are associated with substantial oncological benefit [1–8].

Retrospective analyses suggest that continuation of trastuzumab in the setting of asymptomatic LV dysfunction may be safe with appropriate cardiac management [17, 18]. To date, there is no available prospective data regarding the safety of trastuzumab, pertuzumab and T-DM1 in the setting of cardiac dysfunction. The SAFE-HEaRt study (ClinicalTrials.gov, Identifier: NCT01904903) is the first prospective study to test the hypothesis that trastuzumab, pertuzumab and T-DM1 can safely be used in patients with compromised LV systolic function, along with cardiac monitoring and treatment with cardioprotective agents [19]. This group of patients at present have limited oncologic treatment options, and therefore, are at risk for adverse cancer outcomes.

## Materials and methods

### Trial design

This is a pilot study assessing the cardiac safety of trastuzumab, pertuzumab and T-DM1 in patients with cardiac dysfunction. The trial was conducted at three centers in the U.S with accrual from October 2013 to December 2017. Details of the study design have been previously published [19]. All patients signed informed consent. Data were reviewed by a cardiac review panel composed of 3 board-certified cardiologists with expertise in echocardiography every 3 months and by a data safety monitoring board every 6 months. An Investigational New Drug (IND) Application was obtained from the FDA for trastuzumab, pertuzumab and T-DM1 (IND Number 118811). Minimal duration of planned oncology therapy was 3 months. Patients could be on study for a maximum of 12 months and were followed for 6 months after completion of study treatment. The protocol was amended in 2017 to allow contact with patients for long-term follow up. The study was conducted with approval from the MedStar Health Research Institute (MHRI)-Georgetown University Oncology Institutional Review Board (IRB) and the Memorial Sloan Kettering Cancer Center IRB.

### Eligibility

Participants with stage I-IV breast cancer who were receiving or planning to receive trastuzumab, trastuzumab with pertuzumab or T-DM1 in the (neo)adjuvant or metastatic setting and had LVEF 40 to 49% prior to study participation, were eligible. Baseline LVEF was confirmed by the MHRI Echocardiography Core Laboratory (Core Lab) [19]. Previous HER2-targeted therapies and anthracyclines were allowed; however, the most recent anthracycline administration had to be completed more than 50 days prior to enrollment. Exclusion criteria included symptomatic HF or HF hospitalization for HF within the last 12 months. During screening, all patients were evaluated by study cardiologists to exclude coronary ischemia and/or other treatable causes of HF. Stress testing and coronary artery imaging were performed at the discretion of the study cardiologists.

### Intervention

Patients received HER2-targeted therapy with or without chemotherapy or endocrine therapy as per the treating oncologist’s choice concurrently with the study-mandated cardiac monitoring and cardioprotective medications. Trastuzumab and pertuzumab doses were not reduced. T-DM1 dose was reduced per standard oncologic criteria, but not adjusted for cardiac reasons. Doses of chemotherapy were adjusted as indicated at the discretion of the oncologist.

Cardiac treatment with beta blockers (BB), angiotensin-converting enzyme (ACE) inhibitors (ACEi) or angiotensin II receptor blockers (ARBs) was managed by study cardiologists and initiated in all patients who did not have contraindications prior to the start of on-study HER2-targeted therapy. BB was initiated prior to the first dose of on-study HER2-targeted therapy and subsequently titrated to the maximum tolerated dose. If this was achieved, an ACEi was added and increased as tolerated to the maximum dose. The algorithm detailing the cardiac medication titration has been previously published [19]. All patients were given home blood pressure (BP) cuffs to monitor daily BP to facilitate dose titration. Carvedilol was the preferred BB and was initiated first, followed by ramipril (ACEi) or candesartan (ARB). Patients who were previously on a different BB were changed to carvedilol; however, other ACEi or ARBs were allowed and continued if they were part of patient’s medications prior to the trial.

Cardiac assessments included echocardiograms and cardiology visits at baseline, 6 weeks and 12 weeks and then every 12 weeks while on study. Echocardiograms were repeated at the end of treatment (EOT) and 6 months post EOT. All echocardiograms were acquired following a study-specific protocol and were independently reviewed by the Core Lab, blinded to any clinical information. LVEF was assessed by 3D, 2D biplane Simpson’s method and visual estimate following American Society of Echocardiography guidelines [20]. All available LVEF values were included on the case report form, sent to the investigators and used for clinical decision-making, following a hierarchical approach:

1. if 3D measurement LVEF was available it was used as LVEF of record for that study;
2. if 3D measurement was not available, 2D LVEF was used; and
3. if neither 3D or 2D measurements were available, visual estimated LVEF was used.

Protocol defined recommendations regarding interruption of HER2-targeted therapies based on LVEF were followed [19]. If there was an asymptomatic absolute decline in LVEF of > 10% points from baseline or to ≤ 35%, HER2-targeted therapy was temporarily withheld. This prompted a cardiology assessment of signs and symptoms of HF and a confirmatory study echocardiogram in 2–4 weeks. If repeated study echocardiogram confirmed LVEF decline, the patient came off the study and the event was considered an asymptomatic worsening of LVEF. However, if the repeated echocardiogram showed improvement in LVEF and the holding criteria were no longer met, HER2-targeted therapy was resumed. If a patient developed symptomatic HF at any time confirmed by the study cardiologist, the patient went off study and the event was considered a CE. Interruptions in HER2-targeted therapies according to the study algorithm did not affect chemotherapy and endocrine therapies and decisions regarding continuation of those were at the discretion of the treating oncologist.

### Study endpoints

The primary endpoint was the proportion of patients who completed planned HER2-targeted therapy without developing asymptomatic worsening of cardiac function or CE, defined as symptomatic HF; cardiac arrhythmia requiring pharmacological or electrical treatment; myocardial infarction; sudden cardiac death or death due to myocardial infarction, arrhythmia or HF. Asymptomatic worsening of cardiac function was defined as asymptomatic decline in LVEF > 10% points from baseline and/or LVEF ≤ 35% corroborated by a confirmatory echocardiogram 2–4 weeks and was not considered a CE. Planned HER2-targeted therapy was defined according to the treatment intent. In the (neo)adjuvant setting, planned therapy was considered completion of 1 year of trastuzumab with or without pertuzumab. If a patient had already completed part of the 1 year (neo)adjuvant course prior to enrollment in the trial, the planned oncology therapy course was defined as the time at which 1 year of HER2-targeted therapy was completed (with only a portion of this occurring on study). In the metastatic setting, planned HER2-targeted therapy course was defined as 1 year of treatment or the time until which cessation of the treatment regimen occurred due to non-cardiac toxicity or progressive disease requiring change in oncologic therapy.

### Statistical methods

The categorical data analysis methods were used for primary analysis of planned HER2-targeted therapy completion without a CE or asymptomatic worsening of cardiac function. An exact confidence interval of the treatment completion rate based on binomial calculation was obtained. The longitudinal generalized linear model was used to assess the LVEF measures and take CE or asymptomatic LVEF decline into consideration. Wilcoxon rank-sum test was used to compare absolute changes in LVEF from baseline to each time point between those with and without cardiac dysfunction. No imputation was done for patients with missing data. Statistical analysis was performed using SAS 9.4 and graphics were generated by RStudio version 0.99.902 and Excel.

### Sample size calculation

We estimated that at present only 10% of patients with HER2-positive breast cancer and reduced LVEF receive HER2-targeted therapy. We proposed that if at least 30% of study participants could complete their planned HER2-targeted therapy on study, this would represent a clinically meaningful increase in the proportion of patients receiving a therapy with a substantial oncologic benefit. Therefore, we defined a completion rate of planned oncology therapy of 30% as clinically relevant, and a completion rate of 10% as similar to current practice. A sample size of 30 patients was planned based on the primary endpoint. A two-stage design with an interim analysis for safety after 15 patients were enrolled was planned with 80% power at a significance level of 5% with two-sided *P-*value. Safety rules also included planned early trial termination in the setting of any cardiac death or three patients experiencing a CE.

## Results

### Patient characteristics

**Fig. 1:**
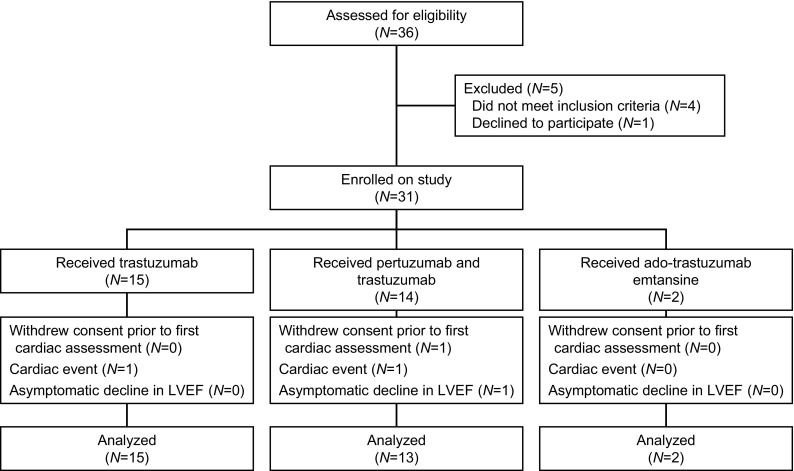
Patient disposition on the SAFE-HEaRt study

In total, 36 patients with HER2-positive breast cancer and cardiac dysfunction were screened, 31 patients were enrolled and 30 patients underwent at least one echocardiogram while on study (Fig. 1). One patient withdrew consent after receiving one dose of HER2-targeted therapy on study due to personal reasons. Because she did not complete at least one follow-up echocardiogram while on study, this patient was not included in the analysis. The patients’ characteristics are described in Table 1. Mean age at enrollment was 53.6 (± 12.5) years. Most patients (*N* = 28) experienced LVEF decline during HER2-targeted therapy that preceded study enrollment. In 10% (*N* = 3) of patients LV dysfunction was identified prior to the initiation of HER2-targeted therapy.

**Table 1:** Patient demographics

Variable		N (%)
Race/ethnicity		
Non-Latino black		17 (54.8)
Non-Latino white		14 (45.2)
Hispanic/Latino		2 (6.7)
BMI		
Normal (18.5 to < 25)		5 (16.1)
Overweight (25 to < 30)		10 (32.3)
Obese (> 30)		16 (51.6)
Hypertension		13 (41.9)
Diabetes		5 (16.1)
Dyslipidemia		12 (38.7)
Prior radiation therapy		11 (35.5)
Prior anthracyclines		17 (54.8)
Prior HER2-targeted therapy		
T		12 (38.7)
T + P		17 (54.8)
T-DM1		2 (6.4)
Breast cancer stage		
I-III		18 (58.1)
IV		13 (41.9)
HER2-targeted therapy received on study		
T		15 (48.4)
T + P		14 (45.2)
T-DM1		2 (6.4)
Beta blockers on study	Yes	27 (90)
ACEi/ARBs on study	Yes	21 (70)

*BMI* body mass index, *T* trastuzumab, *T* + *P* trastuzumab and pertuzumab, *T-DM1* ado-trastuzumab emtansine, *ACEi* angiotensin converting enzyme (ACE) inhibitors, *ARBs* angiotensin II receptor blockers

Participants received an average of 11 cycles (range 7–16) of HER2-targeted therapies while on study. Most patients did not receive chemotherapy while on study (63.3%). Regarding cardiac medications, 27 patients (90%) received carvedilol while bradycardia and severe asthma precluded the use of BB in 2 and 1 patients, respectively. Twenty-one patients (70%) received an ACEi or an ARB. In most patients (17/27, 63%), the maximum tolerated carvedilol dose was less than maximal recommended and dose increases were limited by bradycardia. Similarly, for ACEi and ARBs, most patients were not on maximum recommended doses due to relative low BP on home monitoring. The most common AEs reported during study treatment were fatigue and neuropathy, majority being grade 1–2 (Supplementary Table 1).

### LVEF results and cardiac events

**Fig. 2:**
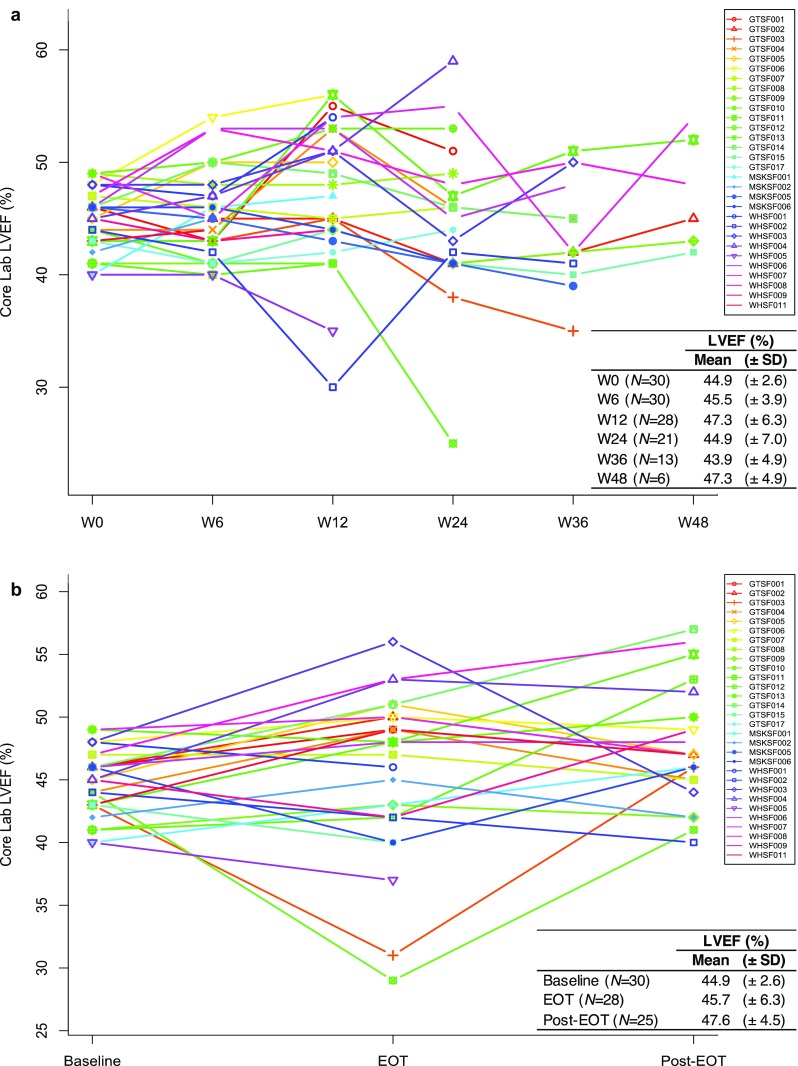
**a** Left ventricular ejection fraction (LVEF) variations during treatment. **b** LVEF at baseline, end of treatment (EOT) and 6 months later (Post-EOT). Abbreviations: *W0* week 0, *W6* week 6, *W12* week 12, *W24* week 24, *W36* week 36; *W48* week 48, *SD* Standard deviation

Mean LVEF measurements at baseline, 6 weeks, 12 weeks and every 12 weeks thereafter, EOT and 6 months post-EOT are shown in Fig. 2. Overall, there were no differences in mean LVEF at baseline (44.8% ± 2.6) compared to EOT (45.7% ± 6.3) and 6-month post EOT (47.6% ± 4.5). Nine patients (33.3%) with metastatic disease continued HER2-targeted therapies beyond study participation. There was no difference in the LVEF 6 months post EOT between patients who remained on HER2-targeted therapies after study completion and patients who did not (*P* = 0.22) (Supplementary Table 2).

**Fig. 3:**
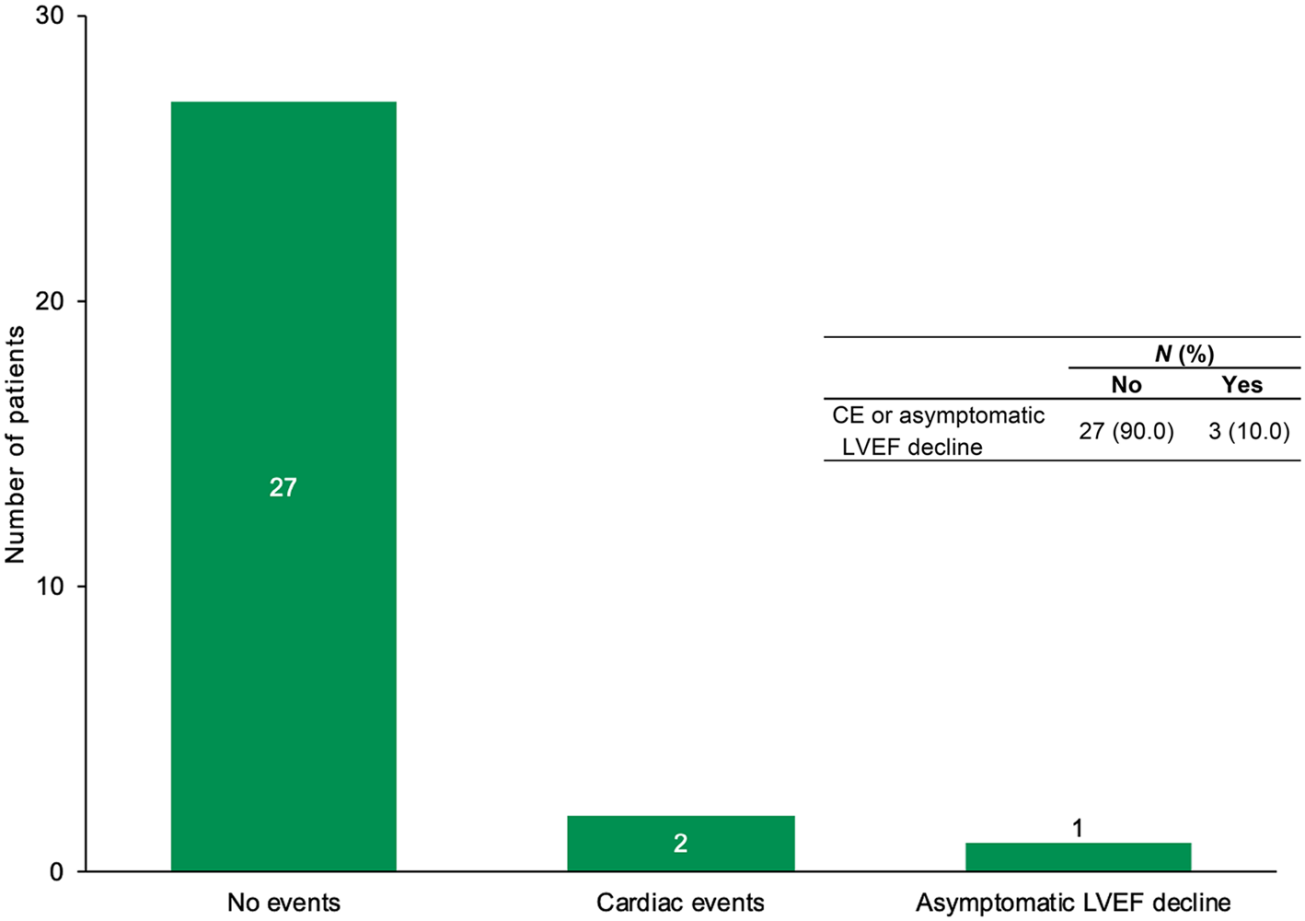
Cardiac event (CE) or asymptomatic decline of left ventricular ejection fraction (LVEF) in the study population

Twenty-seven patients (90%) completed their planned HER2-targeted therapies without developing a CE or protocol-defined asymptomatic decline in LVEF (two-sided 95% CI 73.4–97.9%). Of the three patients who did not complete their planned oncologic therapy, 2 developed symptomatic HF, meeting criteria for a CE, and 1 had a protocol defined asymptomatic LVEF decline to 32% (Fig. 3). All three were taken off study. One of these three patients is alive and continues follow-up and two died due to disease progression at 5 and 16 months after the last study treatment (Supplementary Table 3). Patients who developed a CE or asymptomatic LVEF decline were on study on average 229 days. Baseline LVEF was not different between patients who developed a CE or asymptomatic LVEF decline and those who did not (*P* = 0.1); however, median LVEF was significantly lower at weeks 6, 24 and EOT among those who developed a CE or asymptomatic decline in LVEF compared to those who did not (*P* < 0.05) (Table 2). There were no cardiac deaths on study. We performed univariate analyses to examine factors associated with development of a CE or asymptomatic decline in LVEF. Age, co-morbidities, prior anthracyclines or radiation, cancer stage, type of HER2-targeted therapy and cardiac medications on study were not associated with CE.

**Table 2:** Left ventricular ejection fraction (LVEF) variations according to the development of a cardiac event (CE) or protocol defined decrease in LVEF (Wilcoxon rank-sum test)

Time point	Median LVEF (assessed by core lab), % [IQR]			*P* value
	Overall study population (*N* = 30)	No events (*N* = 27)	CE or asymptomatic decline in LVEF (*N* = 3)	
Baseline	45.0 [43, 47]	46.0 [43, 47]	43.0 [40, 44]	0.10
6 weeks	45.0 [43, 47]	45.0 [43, 48]	41.0 [40, 43]	0.04
12 weeks	47.5 [44, 53]	49.0 [44, 53]	41.0 [35, 45]	0.07
24 weeks	45.0 [41, 48]	46.0 [41, 49]	31.5 [25, 38]	0.04
End of treatment (EOT)	48.0 [42, 50]	48.0 [43, 50]	31.0 [29, 37]	0.01
6 months post-EOT	47.0 [45, 50]	47.0 [45, 50]	43.5 [41, 46]	0.16

*IQR* interquartile range

## Discussion

The SAFE-HEaRt trial is the first prospective study to demonstrate safety of HER2-targeted therapies in patients with reduced cardiac function defined as LVEF between 40 and 49% with concomitant treatment with carvedilol and renin-angiotensin inhibitors. The trial met its primary endpoint with 27 patients (90%) completing planned oncologic therapy without developing a CE or asymptomatic decline in LVEF, with a two-sided 95% CI 73.4–97.9%. These results support the concept that through cardiology and oncology collaboration, this patient population can receive optimal cancer therapy while minimizing the risk of poor cardiac outcomes.

The survival impact of withholding or delaying HER2-targeted therapies on oncologic outcomes is not well defined. Current FDA approved package inserts for trastuzumab, pertuzumab and T-DM1 recommend holding these drugs in the presence of cardiomyopathy pre-treatment or LVEF decreases during treatment as follows: absolute decrease in LVEF ≥ 16% from pre-treatment values or LVEF ≤ 50% and ≥ 10% absolute decrease from baseline (trastuzumab); LVEF < 40% or LVEF of 40–45% with 10% or greater absolute decrease below pretreatment values (pertuzumab and T-DM1) [14–16]. Interestingly, there is no prospective data supporting the different cut-offs. In the SAFE-HEaRt study, after careful discussion with cardiologists and oncologists, the cut-off to hold therapy was further lowered. According to the current package inserts, most patients enrolled in this study would have required interruption in HER2-targeted therapies until LVEF recovery or discontinuation of therapy.

The SAFE-HEaRt study aimed at enrolling “real world patients” with racial and ethnic diversity (over 65% of patients self-identified as non-Latino Black or Latino) and a high incidence of co-morbidities including advanced age, hypertension and previous receipt of anthracyclines [9–11]. Indeed, the participants in the SAFE-HEaRt study fall into the category of “high risk” based on the American Society of Clinical Oncology Clinical Practice Guideline for Prevention and Monitoring of Cardiac Dysfunction [21] and our study provides a model for successful co-management of patients by the oncologists and cardiologists. Recent studies reported benefit of ACEi and/or BB as primary prevention strategy in patients with normal LVEF during treatment with epirubicin [22] or trastuzumab [23], however, these trials excluded patients with lower LVEF who may potentially have the greatest benefit from this type of cardiac intervention. Another strength of our study is the central LVEF assessment by the Core Lab. The importance of core laboratory cardiac imaging is well known in cardiology research given differences among imaging and has only recently been investigated in oncology clinical trials [24, 25].

The limitations of this trial are the small sample size, lack of randomization and the heterogeneity of the study population. The fact that the study was conducted at three large centers with both cardiology and oncology expertise may limit its generalizability in centers without readily available expertise in cardio-oncology. Future steps include long-term follow-up and larger implementation studies in the community setting.

In summary, the SAFE-HEaRt is the first study to provide prospective data on the safety of the use of HER2-targeted therapies in patients with breast cancer and compromised cardiac function. The results provide the basis for clinical practice changes supporting the use of HER2-targeted therapies in selected asymptomatic patients with LVEF between 40–49% in close collaboration with cardiology. This conclusion is particularly important given the major advance that HER2-targeted therapies constitute and the potential negative impact on survival of delaying or discontinuing these therapies.

## Electronic supplementary material

Below is the link to the electronic supplementary material.

Supplementary material 1 (DOC 58 KB)

## Data Availability

The datasets during and/or analyzed during the current study are available from the corresponding author on reasonable request.
