# Supplementary material for: Prospective evaluation of the cardiac safety of HER2-targeted therapies in patients with HER2-positive breast cancer and compromised heart function: the SAFE-HEaRt study
Source: Breast Cancer Res Treat. 2019 Mar 9;175(3):595–603. doi: 10.1007/s10549-019-05191-2 (PMC6534513; doi:10.1007/s10549-019-05191-2)
Supplement: Supplementary file 1 — Supplementary material 1 (DOC 58 KB) [file 10549_2019_5191_MOESM1_ESM.doc]

**Electronic Supplementary Material**

*Breast Cancer Research and Treatment*

**Prospective evaluation of the cardiac safety of HER2-targeted therapies in patients with HER2-positive breast cancer and compromised heart function: the SAFE-HEaRt study**

F. Lynce, A. Barac, X. Geng, C. Dang, A.F. Yu, K. L. Smith, C. Gallagher, P.R. Pohlmann, R. Nunes, P. Herbolsheimer, R. Warren, M.B. Srichai, M. Hofmeyer, A. Cunningham, P. Timothee, F.M. Asch, A. Shajahan-Haq, M.T. Tan, C. Isaacs, S.M. Swain^*^

***^*^****Georgetown Lombardi Comprehensive Cancer Center,* [*sandra.swain@georgetown.edu*](mailto:sandra.swain@georgetown.edu)

**Supplementary Table 1** Summary of adverse events among patients during treatment on study

| **Adverse events (*N*=31)** | **Any Grade, *N* (%)** | **Grade 3, *N* (%)** | **Grade 4, *N* (%)** |
| --- | --- | --- | --- |
| Anemia | 2 (6.5) | 1 (3.2) | 0 (0.0) |
| Alkaline phosphatase increased | 3 (9.7) | 0 (0.0) | 0 (0.0) |
| Anorexia | 2 (6.5) | 0 (0.0) | 0 (0.0) |
| Arthralgias | 3 (9.7) | 0 (0.0) | 0 (0.0) |
| Blood bilirubin increased | 2 (6.5) | 1 (3.2) | 1 (3.2) |
| Blurred vision | 3 (9.7) | 0 (0.0) | 0 (0.0) |
| Diarrhea | 7 (22.6) | 1 (3.2) | 0 (0.0) |
| Fatigue | 16 (51.6) | 1 (3.2) | 0 (0.0) |
| Hypokalemia | 2 (6.5) | 1 (3.2) | 0 (0.0) |
| Infections | 8 (25.8) | 3 (9.7) | 1 (3.2) |
| Myalgias | 1 (3.2) | 1 (3.2) | 0 (0.0) |
| Nausea | 8 (25.8) | 0 (0.0) | 0 (0.0) |
| Neuropathy | 13 (41.9) | 0 (0.0) | 0 (0.0) |
| QT prolongation | 1 (3.2) | 0 (0.0) | 0 (0.0) |
| Rash | 6 (19.4) | 0 (0.0) | 0 (0.0) |

**Supplementary Table 2** Differences in core left ventricular ejection fraction (LVEF) according to further receipt of HER2-targeted therapy off study, in patients without cardiac event or protocol-defined asymptomatic drop in LVEF only (Wilcoxon rank-sum test)

| **Time point** | **Mean (± SD)[[1]](#footnote-2) LVEF (assessed at Core Lab), %** | | ***P-*value** |
| --- | --- | --- | --- |
|  | **Continued HER2-targeted therapy after study participation (*N=*9)** | **No further HER2-targeted therapy after study participation (*N=*18)** |  |
| Baseline | 45.33 (± 3.12) | 44.89 (± 2.25) | 0.7002 |
| End of treatment (EOT) | 47.78 (± 5.02) | 45.12 (± 6.85) | 0.6494 |
| 6 months post-EOT | 49.38 (± 5.07) | 46.82 (± 4.05) | 0.22 |

**Supplementary Table 3** Description of cardiac and protocol-defined asymptomatic decline in left ventricular ejection fraction (LVEF) events that occurred on study

|  | **Description** | **Prior anthracycline/**  **Co-morbidities^*^** | **LVEF baseline/ lowest/ last-known** | **Time on study before event/**  **On study doses HER2-targeted therapy** |
| --- | --- | --- | --- | --- |
| **CE[[2]](#footnote-3) (Symptomatic HF[[3]](#footnote-4) at 36 wks)** | 28 y/o on adjuvant T[[4]](#footnote-5); s/p R breast reconstructive surgery w/ chest wall fat grafting. Developed signs of HF after surgery; resolved w/ diuresis. | Yes / No | 43% / 31% / 46% (6-mo. f/u) | 257 days / 12 doses |
| **Asymptomatic decline in LVEF** | 61 y/o female on T/P[[5]](#footnote-6) for metastatic disease. Never developed signs of HF. Expired 5 mos. after coming off study due to disease progression. | No / Yes | 40% / 32% / (Expired prior to f/u) | 105 days / 6 doses |
| **CE (Symptomatic HF at 24 wks)** | 62 y/o, hx of lymphoma 35 yrs prior, received anthracyclines and mantle cell radiation. Received T/P + tamoxifen for metastatic disease on study. Developed signs of HF w/o identifiable trigger. Cardiac tx included diuretics and continued HF therapy (candersartan + carvedilol). Expired 16 mos. after coming off study due to disease progression. | Yes / Yes | 46% / 26% / 41% (12-mo. f/u) | 188 days / 8 doses |

1. SD, Standard deviation [↑](#footnote-ref-2)
2. ^*^ Co-morbidities included hypertension, diabetes or hyperlipidemia

   ? CE, Cardiac event [↑](#footnote-ref-3)
3. HF, Heart failure [↑](#footnote-ref-4)
4. T, Trastuzumab [↑](#footnote-ref-5)
5. P, Pertuzumab [↑](#footnote-ref-6)
